# The Athlete Gut Microbiome and its Relevance to Health and Performance: A Review

**DOI:** 10.1007/s40279-022-01785-x

**Published:** 2022-11-18

**Authors:** Marcus T. O’Brien, Orla O’Sullivan, Marcus J. Claesson, Paul D. Cotter

**Affiliations:** 1 SeqBiome Ltd., Cork/Fermoy, Ireland; 2 APC Microbiome Ireland, Cork, Ireland; 3 Teagasc Food Research Centre, Moorepark, Fermoy, Ireland; 4 VistaMilk SFI Research, Cork/Fermoy, Ireland

## Abstract

The human gut microbiome is a complex ecosystem of microorganisms that play an important role in human health, influencing functions such as vitamin uptake, digestion and immunomodulation. While research of the gut microbiome has expanded considerably over the past decade, some areas such as the relationship between exercise and the microbiome remain relatively under investigated. Despite this, multiple studies have shown a potential bidirectional relationship between exercise and the gut microbiome, with some studies demonstrating the possibility of influencing this relationship. This, in turn, could provide a useful route to influence athletic performance via microbiome manipulation, a valuable prospect for many elite athletes and their teams. The evidence supporting the potential benefits of pursuing this route and associated future perspectives are discussed in this review.

## Key Points

**Table:** 

Numerous studies have reported differences between the gut microbiomes of athletes and non-athletes, with health-associated bacteria often being positively associated with physical activity.
Specific pro-/pre-biotics have been shown to positively affect a variety of performance metrics in both animal models and humans, although the mechanisms are still poorly understood.
Athletes can suffer from gastrointestinal and respiratory infections as a consequence of intense exercise. Multiple studies have shown that the microbiome composition may play a role in the gastrointestinal and respiratory infections that athletes suffer as a consequence of intense exercise.

## Setting the Scene

The gut microbiome consists of approximately 40 trillion microbial cells [1] and, thanks to insights provided by high throughput sequencing and culture independent technologies, there is now evidence to suggest that the human gut microbiome plays an important role in immunomodulation, digestion, vitamin metabolism, mood regulation, and a variety of other key functions [2–4]. The associated enhanced interest in the field has been reflected in a ten-fold increase in the number of manuscripts published on the human microbiome from 2010 to 2018 [5].

The human gut microbiome, defined as the collective genomes of microbiota residing within the gut, can be influenced by a variety of factors, such as medication, age and diet [6–8]. The vital importance of the gut microbiome, and its considerable plasticity relative to our human genomes, makes it uniquely suited for modulation to improve human health and performance. While gut microbiome composition is generally stable over time, certain factors can drastically impact the composition, with equally drastic effects on health [9].

In recent years, interest in the importance of the gut microbiome in athletic performance has been slowly growing, with a significant number of studies being dedicated to investigating the potential ergogenic effects of the gut microbiome and microbiome-modifying treatments [10]. While currently, no clear recommendations exist surrounding microbiome-modifying treatments for athletes, it seems likely that there is a substantial amount of untapped potential with regard to the gut microbiome and athletic performance [11]. This potential, and quickly building interest, indicates that understanding and utilising the gut microbiome may become a vital part of sports nutrition in the future, and as such, the bidirectional relationship between the gut microbiome and athletic performance is the main focus of this review.

## The Bidirectional Relationship Between the Microbiome and Exercise

**Fig. 1:**
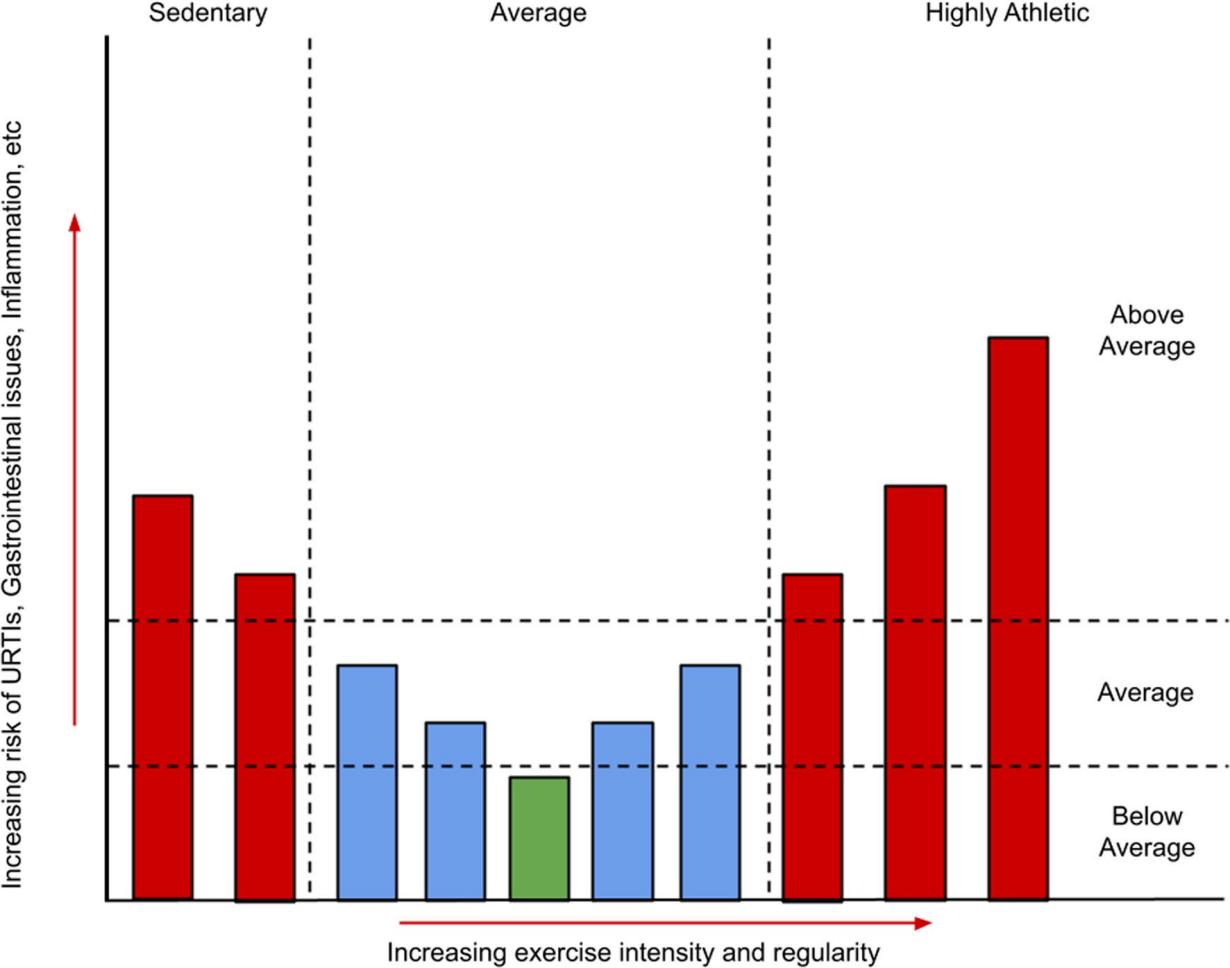
A graph illustrating the hypothesised hormetic effect of varying degrees of exercise on gut health and immunity. *URTIs* upper respiratory tract infections

It has long been observed that exercise can have a profound effect on gut health, and indeed has been proposed as a treatment for a variety of chronic gastrointestinal diseases [12]. Exercise is believed to have a “J-curve” shaped, or hormetic, effect on gut health and immunity, with a moderate amount of exercise having a positive effect on addressing issues related to gut permeability and inflammation, while intense and sustained exercise can have a deleterious effect [13–15], evidenced by the fact that elite endurance athletes often complain of a variety of gastrointestinal disorders during or after exercise (Fig. 1). In one study, 96% of the participants in a 161 km ultramarathon experienced some form of gastrointestinal symptoms during the race (e.g., belching, nausea, vomiting), with 35.6% attributing their failure to finish the race to these symptoms [16]. On the other extreme of the spectrum, one study demonstrated that serum endotoxin levels were significantly higher in sedentary individuals with normal body composition compared to those of trained cyclists, supporting the theory that exercise has a hormetic effect on gut health [17]. The negative symptoms associated with strenuous exercise are believed to be primarily due to a redistribution of blood causing a lack of blood flow to the gut, known as intestinal ischaemia [18, 19], which in turn leads to increased gut inflammation and permeability [20]. Intestinal ischaemia, along with any reperfusion damage caused as blood flow is restored to the gut, may contribute to longer-lasting conditions such as gastritis or ulcers [21]. While intestinal ischaemia was previously thought to be largely the sole contributor to exercise-induced gastrointestinal issues, evidence of a role for the gut microbiome is accumulating. Indeed, in one instance it was shown that germ-free mice or mice treated with specific probiotics were resistant to gut ischaemia-related issues [22]. Probiotics are defined as “live microorganisms, which when administered in adequate amounts, confer a health benefit on the host” [23]. Additionally, various studies show that supplementation with particular strains of probiotics pre-race can have protective effects against gastrointestinal distress and upper respiratory tract infections (URTIs) in athletes, although the mechanisms are not yet fully understood [24–26].

### The Microbiome, Respiratory Tract Infections and Inflammation

A common problem affecting elite athletes, due to the aforementioned immunosuppressive effect of exercise at high intensity, is an increased incidence of URTIs that can have significant negative impacts on training and performance [27].

Multiple studies have shown that specific probiotics can have a significant protective effect against URTIs, possibly due to specific immunomodulatory properties [28]. Tavares-Silva and co-authors found that a probiotic blend (*Lactobacillus acidophilus* LB-G80*, Lacticaseibacillus paracasei* LPc-G110*, Lactococcus lactis* LLL-G25*, Bifidobacterium animalis* subsp. *lactis* BL-G101, and *Bifidobacterium bifidum* BB-G90), showed a significant reduction in URTI symptoms and a 29% reduction in URTI incidents in marathon runners [26]. This was affirmed by another study, which utilised a probiotic blend (*B. bifidum* W23*, Bifidobacterium lactis* W51*, Enterococcus faecium* W54*, Lb. acidophilus* W22*, Levilactobacillus brevis* W63, and *L. lactis* W58) leading to the observation of a 2.2-fold decrease in URTI symptoms in the probiotic group compared to the placebo, alongside a reduced exercise-associated drop in tryptophan levels, which is believed to be an important factor in exercise-induced immunosuppression [29].

Furthermore, URTI prevalence, along with gastrointestinal issues, is thought to be closely related to levels of chronic systemic inflammation, believed to be partly caused by the increased permeability of the gut associated with intense exercise. This allows for pro-inflammatory molecules, such as bacterial lipopolysaccharides, to enter circulation, causing the aforementioned chronic low-grade inflammation [30]. To counteract this, research has shown that multiple probiotics can lessen this inflammatory response in a variety of ways, potentially mediating the negative knock-on consequences, reducing gastrointestinal symptoms and URTI occurrence/severity [11]. This indicates that anti-inflammatory pre-/probiotics may be directly beneficial in reducing the performance loss associated with chronic inflammation. Additionally, there may be an indirect benefit arising from a reduced need for standard anti-inflammatory drugs, which are known to have significant impact on the gut microbiome [31].

As such, these observations show that there is promise in employing specific probiotics to reduce URTI incidence and inflammation, minimising their impact on performance.

### The Interplay Between the Gut Microbiome and Athleticism

While the impact of the gut microbiome on inflammation is important to note, it must be considered that the interaction between exercise and the gut microbiome is not one directional. It has been well established that there are considerable differences in microbiome composition and diversity between athletes and sedentary individuals, with athletes tending to exhibit higher levels of alpha diversity and various health-associated microbiota [30]. Studies have noted that these differences may be both predictive and impacted by athletic performance, as discussed further in this section.

First, it is important to consider that not all exercise is equal, with some studies suggesting that there are significant microbiome differences between athletes competing in different types of sports. Sports can be broadly categorised according to either their static or dynamic requirements. Static sports are those which involve intramuscular forces, measured by maximal voluntary contraction, and are associated with muscular strength (e.g., weightlifting). Dynamic sports, on the other hand, involve changes in muscle length and joint movement, measured in maximal oxygen consumption, and associated with cardiovascular fitness (e.g., long-distance running) [32]. The combination of these measurements can be used to generate sports-group classifications. O’Donovan and colleagues observed differences in the relative abundance of health-associated bacteria across nine different sports-group classifications in elite athletes, with sports being classified as either low, medium or high with regard to both static and dynamic components. This included an enrichment of species of *Bifidobacterium, Lactobacillus, Prevotella* and *Faecalibacterium* in the gut of athletes competing in sports with a high dynamic component and low static component [33]. A related study found that microbiome composition was predictive of exercise gains in both resistance and cardiovascular exercise, with the taxa that were significant in this regard differing between the two modalities of exercise, and cardiovascular exercise having a minor, transient effect on microbiome composition [34].

With regard to dynamic/endurance-based sports, studies have shown that certain probiotics can have positive effects on an athlete’s endurance. One study observed a significant increase in both grip strength and swim-to-exhaustion time of mice supplemented with *Lactiplantibacillus plantarum* TWK10 extracted from fermented cabbage [35]. The mechanism of performance improvement is poorly understood, although the researchers observed significantly lower lactate levels in mice treated with probiotics, and commented on the fact that *Lactobacillus* supplementation has been known to reduce atrophy markers and muscle turnover in previous studies, indicating that this may be the performance-enhancing mechanism at play; however, as with all animal studies, it is unclear if these benefits would remain when applied to human subjects.

Scheiman et al. published perhaps one of the most influential studies on the topic in which the species *Veillonella atypica* was noted to be highly enriched in the faecal samples of marathon runners [36]. This agrees with another investigation that noted a dramatic increase in the genera *Veillonella* and *Streptococcus* in the guts of ultra-marathon runners post-race. In addition to this, Scheimann et al. noted that supplementing mice with *V. atypica* isolated from the marathon-runners’ faecal samples significantly improved treadmill performance as opposed to a *Lactobacillus delbrueckii* subsp. *bulgaricus* control. Scheiman et al. pointed to *V. atypica*’s lactate metabolism as a potential basis of any benefits observed, with evidence of multiple lactate utilisation genes being upregulated post-exercise, and demonstrated that radio-labelled lactate could cross the epithelial barrier into the gut, indicating that *V. atypica* could increase lactate turnover and thereby decrease lactic-acid build up in the muscles [36, 37]. However, other papers have pointed out that the use of a *L. delbrueckii *subsp.* bulgaricus* control may have biased the results, as this organism has been shown to produce lactate, and consequently have a negative impact on exercise to exhaustion time [38].

It is also thought that certain microbial metabolites may play important roles in energy production, with one such example being short chain fatty acids (SCFAs), thought to not only to be an important energy source, but also play an important role in gut homeostasis and immunomodulation [30]. Multiple studies have noted an increase in microbial-derived faecal SCFAs in athletes, especially butyrate, relative to controls. In their investigation, Estaki et al. noted that the VO_2_ max of human subjects was strongly correlated with butyrate production, along with associated butyrate-producing bacteria such as *Clostridiales, Roseburia, Lachnospiraceae* and *Erysipelotrichaceae* [39]. Additionally, a paper by Barton et al. noted an increased level of faecal SCFAs along with associated microbial SCFA-producing pathways in professional rugby players, when compared to sedentary controls [40]. Other papers have noted a number of benefits associated with SCFAs with regard to skeletal muscle, including increased carbohydrate uptake, lipid metabolism and fatty acid oxidation [41].

In addition to energy production, the gut microbiome is also thought to have significant impact on skeletal muscle turnover and maintenance, an important factor in all sports but especially so in primarily static sports. One obvious mechanism influencing these functions is protein metabolism.

The role of the gut microbiome in host protein metabolism has been the focus of many studies. While the small intestinal microbiome is likely to play an important role, studies of these microbes is challenging due to the difficulties associated with sourcing samples from the small intestine, making the research in this area somewhat limited. With regard to the large intestine, the majority of proteolytic activity is attributed to *Bacteroides, Propionibacterium, Streptococcus, Fusobacterium, Clostridium* and *Lactobacillus* species [42, 43]. It is thought that this microbial protein metabolism increases amino acid availability for the host and produces useful microbial metabolites such as SCFAs, which, aside from their aforementioned effects, have been observed to have a positive impact on lean muscle mass in certain animal studies [44, 45]. One such study showed that mice treated with antibiotics (reducing gut microbiome diversity) gained less muscle mass following resistance training than untreated mice that undertook a corresponding level of exercise, supporting the theory that a functioning gut microbiome is important in muscle anabolism [46]. In a similar mouse study, protein type was observed to have a relationship with the microbiome and weight gain. Mice that consumed large amounts of a higher-quality protein (whey) exhibited positive changes in the microbiome (increases in *Akkermansia* and *Bacteroides uniformis* abundances) along with decreased weight gain in response to fat consumption, when compared to mice who consumed a lower-quality protein (casein). Furthermore, when a faecal microbiome transplant was performed between the two groups, the high-quality protein microbiome transferred some of its benefits to its recipients [47]. While these studies are encouraging, animal studies may not always translate into actionable insights for humans, and therefore the results should be interpreted with caution.

Another potential avenue through which the gut microbiome may influence muscle metabolism is via certain nutrient-sensitive pathways such as the mammalian target of rapamycin (mTOR) or AMP-activated protein kinase (AMPK). Both mTOR and AMPK are nutrient-sensitive master regulators of cell metabolism, modulating a variety of homeostatic functions such as ketogenesis, muscle catabolism/anabolism and lipogenesis [48, 49]. The mTOR is a subunit of two protein complexes, mTOR complex 1 (mTORC1) and mTOR complex 2 (mTORC2), which regulate various vital bodily functions such as metabolism, cell survival, protein synthesis and cytoskeletal organisation [50]. The mTOR activity is modulated via a variety of signals such as amino acid, oxygen or stress levels [51]. These signals are communicated to the mTOR pathway via closely related systems, such as AMPK, or growth factors such as insulin-like growth factor 1 (IGF-1). Insulin-like growth factor 1 is the major mediator of prenatal and postnatal growth, influencing growth in bone, cartilage, nerves and, most relevant to athletes, muscle, partially mediated through its interaction with the mTOR pathway [52, 53]. Due to their nutrient sensitive natures, both pathways are influenced by intestinal contents, and by extension, the gut microbiota. This has been demonstrated elegantly by showing that a faecal microbiome transplant from AMPK knock-out mice (which have markedly higher lipid deposition than wild-type mice), transfers some of these effects to healthy mice, clear evidence of bidirectional relationship mediated by this pathway [54]. A similar study showed that mice treated with an mTORC1 inhibitor and fed a high-fat diet had a notably higher abundance of *Akkermansia muciniphila,* a mucin-degrading gut bacteria generally associated with healthy gut microbiomes, than mice fed only a high-fat diet [55]. AMP-activated protein kinase may partially modulate this effect through macrophage polarisation. Macrophage polarization refers to the activation of circulating macrophages into a pro-inflammatory phenotype (M1) or an anti-inflammatory phenotype (M2) in response to various stimuli [56]. Studies have observed that AMPK promotes macrophage proliferation to the M2 phenotype, with an increase in M2 macrophages in turn being associated with a sedentary lifestyle [57, 58]. The mTOR pathway has also been noted to be a crucial factor in muscle synthesis, with one important avenue being the influence of IGF-1. Insulin-like growth factor 1 is the major mediator of prenatal and postnatal growth, influencing growth in bone, cartilage, nerves and various other tissues [53].

Additionally, multiple studies have shown that specific strains of *Weizmannia coagulans* (previously *Bacillus coagulans*) can have measurable effects on protein absorption and metabolism. One study showed significantly increased serum levels of important amino acids post-ingestion of milk with *W. coagulans* as opposed to milk alone [59]. Another study showed that administration of *W. coagulans* pre-training significantly reduced perceived muscle soreness and increased recovery speeds in non-athletes [60]. Supporting this paper, another study found that collegiate athletes showed lower levels of tumour necrosis factor alpha (TNF-α) when supplemented with probiotic *Bacillus subtilis* DE111. Tumour necrosis factor alpha levels in athletes have been associated with negative factors such as stress and disturbed sleep, as well as skeletal muscle protein synthesis [61], indicating that this probiotic may mitigate some of these effects, although no differences in performance metrics were observed in this study. Another study using the same *B. subtilis* strain, given to female collegiate weightlifters, found that participants taking the probiotic experienced greater body fat reductions, although no differences in performance were observed [62].

Taken together, these studies provide strong evidence that the gut microbiome influences multiple functions that are vital to athletic performance. Understanding and exploiting this bidirectional relationship may be a vital aspect of health nutrition in the future.

### Pro-, Pre- and Synbiotics

While understanding the complex interactions between the gut microbiome and athleticism is important, we must consider how we may leverage these understandings to improve current sports nutrition and modulate the gut microbiome in a favourable manner. An obvious pathway to this is through the use of microbiome-modifying substances such as pro-, pre- and synbiotics.

The use of probiotics to positively impact human health has been steadily gaining interest and investment, with the probiotic market estimated to reach a value of 69 billion dollars by 2023 [63]. The market includes various probiotic strains with reported benefits including managing irritable bowel syndrome (IBS) and mood stabilisation [64, 65]. The most common probiotic strains are members of the genus *Bifidobacterium* or the former genus *Lactobacillus* (more recently divided into several new genera), although it is important to note that many of the probiotic effects observed are specific to certain strains [66, 67]. Probiotics are thought to impact the composition of the gut microbiome, and thereby correct potential perturbations, through a variety of mechanisms depending on the exact organism used. Proposed mechanisms include the creation of antimicrobials, competing with harmful microbiota for binding sites, or by modulating intestinal immunity [68].

In addition, various studies have shown an increase in certain athletic-related performance metrics of both animals and humans under microbiome-modifying treatments. One such study was notable in that it found that a four-week supplementation of a mixed-strain probiotic (*Lb. acidophilus, Lacticaseibacillus rhamnosus, Lpb. plantarum, Limosilactobacillus fermentum, B. lactis, Bifidobacterium breve, Bifidobacterium bifidum and Streptococcus thermophilus*) increased athletes’ run-to-fatigue time by an average of 16% on a treadmill test in hot conditions. The mechanism by which endurance was increased was not clear, although a decrease in serum lipopolysaccharide content post-probiotic intervention was noted [69]. Another study showed that when *Lactobacillus salivarius* subspecies *salicinius* SA-03, isolated from the gut microbiome of Olympic-level female weightlifters was administered to mice over a four-week period, it significantly improved swim-to-exhaustion time and grip strength [70].

Another potential method of delivering potentially health-promoting microbes to the gut is through the use of fermented foods. The impact of fermented foods upon gut health in general is well documented, with its origins dating back to 1906 with Elie Metchnikoff’s “The Prolongation of Life”, which suggested that lactic acid bacteria have a positive effect on life expectancy in Bulgarian peasants [71]. Research into fermented foods has developed substantially since then, with multiple papers noting significant changes in the gut microbiome in response to fermented food consumption, including increased abundance of health-associated genera such as *Bifidobacterium* and *Lactobacillus*, and decreases in pathogens such as *Escherichia coli* and *Clostridium perfringens*. Positive changes in health were also noted in association with these microbial changes, such as an increase in SCFA production and decreased bloating [72]. Taken together, these studies indicate that fermented foods can have a significant and positive impact on the gut microbiome and health in general.

Aside from the well-established positive health effects of fermented foods, there is substantially less research conducted on how consumption of such foods could benefit athletes. One paper observed reduced exercise-associated immunosuppression in recreational athletes after an exercise stress test, if they had consumed a fermented milk product containing an unspecified strain of *Lacticaseibacillus casei* [73]. This affirms aforementioned studies by showing a decreased incidence of URTIs when *Lacticaseibacillus*-containing probiotics were used, potentially indicating an alternative method for athletes to achieve the same result.

While research in this specific area is still in its infancy, these studies provide some evidence that specific fermented foods could provide performance benefits to athletes. Additionally fermented foods may be viewed as a more convenient/favourable method of administering health-associated microorganisms.

Another mode of influencing the microbiome is the use of prebiotics, which are defined as “*a substrate that is selectively utilized by host microorganisms conferring a health benefit*” [74]. Examples of prebiotics include fructo-oligosaccharides (FOS), galacto-oligosaccharides (GOS), and polyphenols [75]. These substrates cannot be readily digested directly by humans, but rather are digested by, and result in the enrichment of, certain health-associated microorganisms. Prebiotics may be a fruitful avenue to pursue when aiming to modulate the microbiome and associated biological functions, especially when targeting taxa that would be more challenging to develop as probiotics.

Research into the effects of prebiotics on athlete performance in humans is rather limited and has so far largely only focused on synbiotic supplements. Synbiotics are a combination of probiotics and prebiotics designed to work synergistically to selectively enrich health-promoting microorganisms [76]. While this confounds the specific effects of prebiotics with regard to athletic performance due to the presence of probiotics, the results from synbiotic studies have been encouraging. Roberts et al. showed that the use of a multi-strain probiotic (*Lb. acidophilus* CUL-60*, Lb. acidophilus* CUL-21*, B. bifidum* CUL-20 and *B. animalis subspecies lactis* CUL-34) along with FOS and the antioxidant α-lipoic acid in triathlon runners resulted in a 46.6% reduction in lipopolysaccharide/endotoxin levels (as measured by Limulus amebocyte lysate chromogenic quantification) both pre- and post-race and showed a slight, albeit non-significant, improvement in race times [77]. Another class of prebiotic, polyphenols, are a diverse class of compounds with a common phenolic structure, present in various plant-based foods and their derivatives and observed to have anti-oxidative and anti-inflammatory effects [78]. Sorrenti et al. wrote a comprehensive review of the potential benefits of polyphenols with regard to athleticism, with studies showing various positive effects such as increased muscle recovery, reduced fatigue and reduced lactate production [79]. This review also posits that these effects are likely mediated through the interaction of polyphenol and the gut microbiome, as polyphenols tend to have otherwise poor bioavailability. While it has been demonstrated that polyphenols can impact the gut microbiome, the exact mechanisms through which they may improve athletic performance by doing so still remain unclear.

In combination, these investigations show that, while the associated mechanisms require further investigation, the microbiome-modifying effects of pro-/prebiotics could have a significant impact on the performance of athletes.

### Longitudinal Microbiome Monitoring

It is important to remember that the gut microbiome is a dynamic system that is often in flux, a consideration which should inform gut microbiome research and microbiome-modifying treatment use. As a result, single time-point microbiome profiling often fails to capture the intricacies of the gut microbiome as it changes over time. Longitudinal microbiome monitoring is the concept of using current microbiome profiling technologies to track the changes in an individual's microbiome over time, providing valuable insights on the effects of lifestyle changes on the microbiome and allowing more informed lifestyle choices.

Multiple companies now offer microbiome profiling, although few fully utilise longitudinal analysis. Given the dynamic nature of the gut microbiome, analysis of an individual’s microbiome at just a single time point provides a somewhat limited actionable insight and does not reveal changes arising from a microbiome-modulating intervention. As such, while single time point microbiome monitoring is available as a commercial service, one can suggest that more valuable longitudinal microbiome analysis remains a relatively underutilised tool. Longitudinal monitoring can, for example, provide insights of great value with respect to episodic gastrointestinal diseases, e.g., inflammatory bowel disease (IBD), where significant differences in the gut microbiome have been noted on a timescale of weeks to months [80]. Changes in diet have also been noted to affect the microbiome in a similar fashion, with such changes only being evident over a certain timescale, and have been observed to correlate with certain negative health outcomes [81]. Even more dramatic changes have been observed surrounding gastrointestinal surgery, with one study showing that the difference in the gut microbiome pre- and post-surgery was equivalent to the differences between a marine sediment and rhizosphere microbiome, effects which would be difficult to detect without longitudinal analysis [82]. A study by Levy et al.observed gut microbiome enterotypes, defined as “reproducible patterns of variation in the microbiota”, characterised by a high abundance of either *Bacteroides* or *Prevotella*, with the *Prevotella*-dominated enterotype correlating with certain inflammatory factors, such as TNF-α. Levy et al. also noted that gut microbiomes may occasionally transition between these enterotypes, which are associated with long-term dietary choices [83, 84]. Another example of the value of longitudinal microbiome monitoring relates to the gut microbiome changes observed as a consequence of antibiotic use. It is well known that antibiotic use can have drastic effects on gut health, with the occurrence of antibiotic-associated diarrhoea being one clear example. Antibiotic use can disrupt the normal gut microbiome composition, selecting for certain pathogens such as *Clostridioides difficile*, which in turn leads to gastrointestinal symptoms [85]. However, these symptoms are not always acute; one study showed a drastic reduction in gut microbiome diversity, and a concurrent increase in certain pathobionts, in response to an antibiotic treatment. While the gut microbiome compositions generally returned to levels close to baseline within 1.5 months, certain species remained undetectable after 180 days [86]. This pattern of perturbation before returning to baseline, along with the apparent loss of certain bacterial species, among the other patterns mentioned above, would be missed by single time-point microbiome monitoring. Given that athletes may often require antibiotic treatment or other similarly microbiome-modifying treatments, this information could be highly valuable to them.

Another important factor to consider is gut microbiome stability itself. While the gut microbiome is a dynamic system, healthy gut microbiome composition tends to be relatively stable over time, with large and/or sudden perturbations in composition being associated with disease states or otherwise unhealthy outcomes. Aside from the acute effects of certain factors mentioned above, general gut microbiome instability is an important variable to consider, which would not be observed via single time-point microbiome profiling. In one large-scale study, microbiome instability over five years was associated with certain diseases such as metabolic liver disease and diabetes mellitus, although it is not completely clear if microbiome instability is causative or correlative with these conditions [87]. Gut microbiome stability may also be an important factor in athletic performance, with one study observing that the athletes showing the greatest improvement in performance following a dietary intervention tended to have more stable gut microbiomes [88]. Additionally, in a paper by O’Donovan et al., it was observed that athletes who reported gastrointestinal distress prior to travelling showed greater instability in their gut microbiome compositions [89]. These studies indicate that gut microbiome stability may be an important factor in assessing gut microbiome health and, while generally stable over time, the gut microbiome tends to be most unstable during periods of sudden lifestyle changes or upon exposure to other microbiome-modifying factors, and may lead to or be a biomarker of negative health outcomes. By extension, longitudinal microbiome monitoring would be a valuable tool in measuring gut microbiome stability, which may be an important factor for athletes to consider in the future.

Microbiome monitoring is especially important with regard to athletes, as extreme dietary and lifestyle changes between on and off season could have profound effects on the microbiome and, consequently, performance. By consistent monitoring of the microbiome, it is possible to detect deleterious microbiome shifts and address them before they become problematic, or to monitor an athlete’s gut microbiome response to an intervention. Previous studies have shown that the travel and dietary changes that often accompany the lifestyle of elite athletes can have long-lasting, negative impacts on the gut microbiome [89]. This could potentially be addressed using targeted pro-/prebiotic mixes designed to address any imbalances observed, although a significant amount of additional research is required to effectively develop these technologies and their integration.

## Conclusion

An increasing body of evidence from human and animal studies suggests that exercise has a significant effect on the gut microbiome. This effect appears to generally be positive, increasing gut microbiome diversity and abundance of health-associated bacteria, with some studies suggesting that this is exercise-modality dependent. Developing a further understanding of this relationship and resulting applications would be highly beneficial to both athletes and scientists. In the future it may be possible to simulate the positive effects of long-term exercise on the microbiome using prebiotic, probiotic, diet or other interventions. However, to accomplish this a more granular elucidation of the complex relationship between athletic performance and the microbiome, and the underlying mechanisms, is required.

## Declarations

### Availability of data and material

Data sharing is not applicable to this article as no new data were created or analyzed in this study.
